# Grand Challenges and Opportunities in Surgical Ophthalmology: Together for a Shared Future

**DOI:** 10.3389/fopht.2022.922240

**Published:** 2022-07-04

**Authors:** Yongwei Guo, Vladimir Kratky, Huatao Xie, Xingchao Shentu, Xiaofei Man, Yanling Wang, Wen Wen, Alexander C. Rokohl, Ludwig M. Heindl

**Affiliations:** ^1^ Eye Center, Second Affiliated Hospital, Zhejiang University School of Medicine, Hangzhou, China; ^2^ Zhejiang University Eye Hospital, Hangzhou, China; ^3^ Zhejiang Provincial Key Lab of Ophthalmology, Hangzhou, China; ^4^ Department of Ophthalmology, Queen’s University, Kingston, ON, Canada; ^5^ Department of Ophthalmology, Union Hospital, Tongji Medical College, Huazhong University of Science and Technology, Wuhan, China; ^6^ Department of Ophthalmology, Xinhua Hospital Affiliated to Shanghai Jiaotong University School of Medicine, Shanghai, China; ^7^ Department of Ophthalmology, Beijing Friendship Hospital Affiliated to Capital Medical University, Beijing, China; ^8^ Department of Ophthalmology and Visual Science, Eye and ENT Hospital, Shanghai Medical College, Fudan University, Shanghai, China; ^9^ Department of Ophthalmology, University of Cologne, Faculty of Medicine and University Hospital of Cologne, Cologne, Germany; ^10^ Center for Integrated Oncology (CIO) Aachen-Bonn-Cologne-Duesseldorf, Cologne, Germany

**Keywords:** ocular surface and corneal disease, cataract, glaucoma, retina, strabismus, orbit, plastics, challenge

## Introduction

Surgical Ophthalmology is one of the topical branches of ophthalmology, which strives to relieve patients’ pain through operations. It plays an integral role in modern ophthalmology, involving almost all structures of the eye and its adnexal tissues. Therefore, from every ophthalmic subspecialty, we highlighted some of the biggest challenges and most recent developments in these fields to set the tone of this specialty section, inspire researchers to conduct groundbreaking research, and stimulate ophthalmologists to improve medical practice to serve patients better.

## Ocular Surface

The ocular surface is a complex and crucial component of the visual system. It comprises the cornea, conjunctiva, and adnexa tissues, such as lacrimal glands and meibomian glands. Any disorder in these structures, including corneal ulcers, keratitis, chemical and thermal burns, dry eye disease (DED), etc. falls into the ocular surface disorder (OSD) (1, 2). OSD could severely affect the quality of vision and life and, in severe cases, may lead to blindness. Though with high prevalence, unfortunately, some cases often go undertreated due to the lack of complete understanding of the underlying mechanism of these diseases.

Recent years have witnessed rapid advancement in the researches in the OSD (3, 4). The advances in research and surgical treatment of severe OSDs have shed light on the management of these diseases. Surgical interventions, such as simple limbal epithelial transplantation (SLET) (5, 6) and corneal endothelial transplantation (7), have greatly improved the management of severe OSD. Another area that has lately gained popularity is the treatment of neurotrophic keratitis with corneal neurotization, with excellent outcomes in appropriate cases. It involves the coaptation of a healthy sensory periorbital nerve to the limbal area either directly or *via* an interposed nerve graft (8). There is nearly complete recovery of sensation in most cases, with accompanying improvement of the clinical picture.

We are particularly interested in manuscripts that report the new tools for detecting risk factors and early diagnosis of corneal ectasia, looking to the biomechanical behavior of the cornea and the future research directions for the treatment of the ectatic corneal disorder. Furthermore, potential promising topics include, but are not limited to, discoveries in the mechanisms of different OSDs, potential treatments of different OSDs, novel therapeutic approaches in OSDs, novel surgical treatments in OSDs, consecutive surgical treatment in OSDs, comparisons of the outcomes of different surgical options in OSDs.

## Cataract

Over the last two decades, many new surgery technologies dramatically advanced cataract surgical treatments (9–13). Besides, various molecular studies about cataractogenesis enriched the cataract formation mechanism and provided novel applications for non-surgical treatments, including breakthroughs in possible pharmacological treatments (14–18).

Small incision phacoemulsification cataract surgery with the intraocular lens (IOL) implantation has been a trend in cataract surgery. With the rapid development of the laser, recently, femtosecond laser-assisted cataract surgery (FLACS) has become an important development in the history of cataract surgery. Many clinical studies showed that FLACS offered the perfection of the capsulorhexis and long-term stability of the effective lens position of FLACS, which provided more predictable refractive outcomes (12, 19–21). These were the significant advantages that manual surgery cannot achieve (20). In addition to these practices, FLACS offered a greater precision and repeatability for pre-chop than manual techniques and reduced effective phaco energy (22), especially in hard nuclear cataract surgery (21). Additionally, FLACS offered more precise incisional astigmatism management. As cataract surgery procedures advance every year, patient demands become more challenging. An ‘ideal’ IOL should restore the patients’ vision without glasses or visual compromises at all distances (23). Premium IOLs, including multifocal and accommodative IOLs, were designed to provide clear vision at near and distant focal points without additional spectacle correction and toric IOLs for astigmatism correction (24, 25). With the use of different IOLs, an ‘ideal’ vision can be achieved in some cases at present (26, 27).

To date, the only remedy for cataracts is surgically removing the cloudy lens and substituting it with a suitable IOL. Aiming to find more solutions to cure or prevent cataracts, the studies about cataractogenesis are still a hot topic. Oxidative stress, excess of quinoid substances, aldose reductase activation, and deficiency of autophagy are essential in the progression of cataracts (28, 29). Significant breakthroughs in pharmacological applications appeared in 2015 (30, 31). Novel pharmacological substances, 5-cholesten-3b,25-diol and lanosterol, can reverse lens opacity *via* dissolving the crystallin proteins aggregates. GSH, L-cystine, and rapamycin, as potential drugs, could protect the lens from opacity or reverse the opacity but still need more *in-vivo* testing (18, 32). Notably, the creation and development of high-throughput drug screening platforms, such as lentoid bodies and cataractous animal models (33), allow researchers to find more small molecular applications among millions.

## Glaucoma

There has been an increase in optimizing glaucoma treatment medically and surgically over the past decades, with a subsequent decrease in associated complications, thereby improving the quality of life in glaucoma patients.

For decades, Prostaglandin F2α (PGF2α) analogs have been essential in the treatment of open-angle glaucoma. However, side effects such as prostaglandin-associated periorbitopathy (PAP) seriously affect patients’ treatment persistence. Recently, a new selective prostaglandin-EP2 agonist (omidenepag isopropyl OMDI), which is hydrolyzed to OMD (Omidenepag) when it penetrates the cornea, has shown promising clinical outcomes for decreasing the adverse effects (34). Moreover, clinical trials demonstrated that OMDI was non-inferior to PGF2α analogs in reducing IOP in patients with OHT or POAG and was well tolerated (35).

Although trabeculectomy is currently the gold standard in glaucoma surgery, it has many potential complications. Hence there is a rising interest in various minimally invasive glaucoma surgeries (MIGS) with a lower risk of complications and comparable IOP lower effects with conventional surgeries. According to the different strategies of surgery design, there are two main categories of MIGS available. The first group has yielded promising results in adult and pediatric glaucoma by creating a circumferential incision into SC and reducing the resistance to aqueous outflow (36–39). Another one develops new MIGS devices to provide an alternative pathway through which aqueous humor can effectively exit the anterior chamber, reducing IOP (40–42). MIGS is widely becoming the front-line glaucoma surgery with respect to various advantages (43–48).

Deep learning (DL), a subset of artificial intelligence based on deep neural networks, has made significant breakthroughs in glaucoma image classification and pattern recognition. Studies incorporated with DL for interpreting optical coherence tomography(OCT)or visual field (VF) data have demonstrated exemplary performance for discriminating glaucomatous eyes from normal eyes (49, 50). At the same time, there are currently some virtual reality-based visual field examinations (VR VFs) to check the visual field, which is not only more ergonomic in design but also cost-effective, suggesting that VR perimeters have the potential to examine VFs with high enough confidence, whereby reducing challenges in current perimetry test by providing a more accessible visual field test (51, 52). These high developed techs make “tele-ophthalmology” possible hence enabling patients to communicate with their attending doctors in real-time through the network, which comprehensively promotes the development of remote ophthalmic consultation (53).

## Posterior Eye Segment

The posterior eye segment comprises the back two-thirds of the eye, involving the anterior hyaloid membrane and all of the optical structures behind it: the vitreous humor, retina, choroid, and optic nerve.

The rapid development of fundus imaging technology has led to an increasing range of fundus images, with more accurate picture resolution, non-invasive examination methods, and efficient treatment (54). The range of visual fundus imaging has evolved from 25° to 35°, 45°, and 55° in the early days, and the most remarkable progress has been made with the advent of laser ophthalmoscopy, which has enabled a qualitative leap in the range of fundus imaging, from 55° to over 100°. In particular, ultra-wide-field imaging has enabled fundus imaging to reach even 200° (i.e., to the extent of the vortex vein and the area before the vortex vein). This wider range helps ophthalmologists detect more peripheral fundus diseases and improves the detection rate of disease (55). That was followed by fluorescence angiography, OCT, and OCTA, with resolutions ranging from 10 µm to 5 µm to 3 µm, gradually progressing to the current level of cellular resolution. More accurate examination techniques help ophthalmologists understand the onset and progression of the disease. In recent years, OCTA has been a fascinating new technology, a non-invasive way to quantitatively assess retinal and even choroidal blood flow without invasive fluorescein angiography (56, 57). It is now widely used in fundus vascular disease.

The advent of small-molecule antibodies, represented by anti-VEGF drugs, and the introduction of intravitreal injections have been landmark advances in the treatment of fundus diseases, changing the previous treatment of fundus vascular diseases, reducing the number of vitreous procedures, and significantly improving the prognosis of diseases such as wAMD, macular edema, and diabetic retinopathy. Anti-VEGF drugs have also emerged as a first-line treatment option for many fundus vascular diseases (58). Furthermore, since the advent of standard three-channel 20G vitrectomy in 1972, sutureless vitrectomies of 23G, 25G, and 27G have been introduced, bringing vitrectomy surgery into the minimally invasive era (59, 60). Compared with the traditional 20G three-channel vitrectomy, minimally invasive vitrectomy reduces the size of the scleral incision, dramatically simplifies the surgical procedure, shortens the operating time, and reduces surgical complications. The availability of anti-VEGF drugs and innovations in minimally invasive vitrectomy techniques have improved the cure rate of fundus diseases, reduced surgical complications, and greatly improved the prognosis of patients.

In recent years, with the interdisciplinary interpenetration of molecular biology, cytogenetics, genetic engineering, stem cell biology, and artificial intelligence (61), ophthalmologists’ knowledge and understanding of fundus diseases such as wAMD, diabetic macular edema, and macular hole have gradually improved, and their diagnosis and treatment have also advanced significantly. However, we still do not have effective treatment options for fundus diseases like retinitis pigmentosa and posterior scleral staphyloma in pathological myopia. There is still a need for further clinical and basic research to be conducted in the future.

## Strabismus

Unlike other eye surgery (cataract, glaucoma, and vitreoretinal surgery, among others) in which skilled techniques are the primary factors of successful operation, the key to strabismus surgery is reasonable design. In recent years, several innovations and modifications in strabismus surgery design have been brought up. The innovation in strabismus surgery focused on the merits of techniques in common strabismus and the treatment of paralytic strabismus such as abducens nerve palsy.

The bilateral lateral rectus recession (BLRc) and unilateral lateral rectus recession combined with medial rectus resection (R&R) are the most common two surgical procedures treating basic-type intermittent exotropia (IXT). The Pediatric Eye Disease Investigator Group performed a multicenter, randomized clinical trial in children of 3 to 11 years old to compare the short-term and long-term outcomes. They found no statistically significant difference in the surgical success rates by three years between children treated with both surgeries, respectively (62).

Paralytic strabismus due to cranial nerve palsy is a relatively tricky problem. Strabismus secondary to abducens nerve (CN 6) palsy is the most common paralytic type, and various surgical techniques are now used in the clinic. Hummelshein first introduced vertical rectus transposition (VRT) in 1907 (63, 64), in which full-tendon width superior and inferior rectus with tenotomy transposed to the margins of the lateral rectus. Medial rectus recession would be performed in most cases due to medial rectus contracture (65), but tenotomy of three or more rectus muscles would increase the risk of anterior segment ischemia (ASI). In order to reduce the risk of ASI and also improve abduction ability, VRT procedures were modified to superior rectus transposition (SRT) (66, 67) and vertical rectus belly transposition (VRBT) without tenotomy by Nishida (68) and also modified by Chen Zhao (mVRBT)[50]. The critical step of mVRBT was without tenotomy. The superior rectus belly and inferior rectus belly are transposed to a position 2 mm adjacent and 6–8 mm posterior to the superior and inferior pole of the lateral rectus muscle, resulting in 57.8△ of esotropia correction and 2.3 scales of abduction improvement.

The third nerve (CN 3) palsy treatments are more complicated since the affected branches and their severity vary and may accompany aberrant regeneration. The ideal goal of treating complete CN 3 palsy is to retain alignment at the primary gaze without diplopia. Although previous techniques could improve the deviations in complete CN 3 palsy cases, residual deviations at primary gaze in short-term or long-term follow-up often occur. The recent popular surgery is Gokyigit’s technique (69), The lateral rectus muscle was split with the upper half transposed to the superior border and the lower half to the inferior border of the medial rectus insertion. Since with Gokyigit’s technique, 50% of patients achieved stable alignment, and the other 50% were undercorrected, requiring a second surgery, a modification of the existing technique by force augmentation through the use of equatorial fixation sutures resulting in satisfactory primary gaze alignment in the complete CN 3 palsy (70).

## Ophthalmic Plastic and Reconstructive Surgery

The subspecialty of Ophthalmic Plastic and Reconstructive Surgery has experienced a kind of renaissance for research and innovation over the last 10-15 years. As an overview, we can discuss some of these advancements in terms of medical and surgical approaches to disease management.

On the medical (pharmaceutical) side, probably the most crucial innovation came with the advent of biopharmaceuticals, better known as biologic agents, as a novel targeted treatment for inflammatory conditions of the orbit. That would likely revolutionize the treatment of thyroid eye disease (TED), with drugs such as teprotumumab and tocilizumab showing significant improvement in the condition in recent studies (71, 72). Such targeted treatment with biologics has been the first major innovation in the management of TED in many years. The same can be said for idiopathic orbital inflammatory syndromes (IOIS), where biologics also show great promise (73). Regarding IOIS, advances have also been made to reclassify some of the so-called ‘idiopathic’ inflammatory conditions into specific disease entities, the prime example being the IgG-4 group of diseases (74).

In the field of oncology, targeted molecular therapy has also made a significant leap forward as a treatment option for some cancers, such as advanced, recurrent basal cell carcinoma (BCC), squamous cell carcinoma (SCC), and malignant melanoma of the periorbital tissues, where surgery may not be indicated or feasible. Especially for BCC, vismodegib has been shown to interrupt the abnormally upregulated Hedgehog signaling pathway specifically. This results in significant regression, if not resolution, of the disease (75). Recent advances have also been made with the advent of immune checkpoint inhibitors for extensive and inoperative squamous cell carcinoma (SCC) and metastatic carcinoma. Recently, the PD-1 inhibitor (cemiplimab) has been approved for this indication in the US, and EGFR inhibitors such as cetuximab may also be helpful in some patients (76). Targeted molecular therapy has also attracted research for ocular and adnexal malignant melanomas (77), and secondary orbital tumors, such as lymphomas. In addition to the above-mentioned new treatment options, updated TNM staging protocols and indications for sentinel lymph node (SLN) biopsy/resection have also been added to our management armamentarium in treating periorbital cancers.

Despite anatomy being an old field, advances in the orbital/eyelid region have also occurred. As an example, recent work by MJ Ali on the Horner-Duverney’s muscle microanatomy has added more to our understanding of the lacrimal pump, with the potential existence of a type of canalicular peristaltic function to aid in tear drainage (78). The description of a relatively new finding of a post-aponeurotic fat pad is another example (79).

There has been a steady move towards minimally invasive orbital surgery as far as surgical advancements go (80, 81). Transconjunctival incisions have become relatively common to access anterior orbital tumors, such as the transcaruncular approach to the medial orbit or superior fornix incisions for superior lesions. More work is being done in endoscopic orbital surgery for mid-orbit and the apex (82). Often it is combined with 3D stereo navigation for accurate localization of the deep apical tumor and safer removal. Additionally, custom-made, 3D printed patient-specific implants have been described for orbital fractures and congenital craniofacial anomalies and are being further refined (83).

Minimally invasive surgery has also become the norm in eyelid and lacrimal procedures. Ptosis surgery is now commonly carried out as a minimal incision levator advancement (MILA) or a posterior approach to Muller’s muscle (MMR) and the levator to hide the wound completely. Dacryocystorhinostomies have been done endoscopically for some time, but in the last few years, transcanalicular laser-assisted lacrimal procedures have been studied and are already in use in some countries (84–87).

For surface reconstruction of periorbital defects, bioengineered dermal substitutes have become more popular with advances in autograft, allograft, and xenograft laboratory preparation and the development of entirely synthetic substitutes (88).

Looking at the way forward, the future holds continued refinement and improvement in all areas mentioned above. That is specifically true in the availability and choice of biologic agents as well as the expanding list of conditions to treat. For example, it may well be that primarily thyroid orbitopathy becomes a medical disease, with surgery used only in extreme recalcitrant cases. There will be continued refinement in identifying ‘idiopathic’ inflammations as specific disease entities, which will lend themselves better to targeted systemic treatments. The same will hold for targeted biologic therapy for periorbital and orbital tumors as the primary treatment modality for advanced cases.

Minimal incision and endoscopic orbital surgery would likely become the standard of care for orbital tumors, with the likely innovative expansion to trans-orbital approaches to the middle cranial fossa for neurosurgical cases. Improvement in equipment and laser technology for transcanalicular lacrimal surgery may, in the future, result in a DCR being a 10-minute office procedure (86).

With respect to infections, a recent study described a metagenome analysis of the local lacrimal biome and coined the term ‘lacriome.’ With the aid of DNA sequencing, this technique identifies organisms present well beyond standard laboratory culture methods and may aid in future identifying and studying infectious processes more accurately (89).

Gene therapy has been studied for several years and shows promising results in inherited eye disorders, such as Leber congenital amaurosis and retinitis pigmentosa. More recently, a mouse model of oculopharyngeal muscular dystrophy (OPMD) with a faulty PABPN1 gene was successfully treated with gene therapy, which resulted in regression of muscle aggregates and fibrosis (90). That may hold promise for some inherited diseases in the field of Ophthalmic Plastic Surgery in the future.

Artificial Intelligence (AI) already plays a role in several specialties, such as radiology for routine screening of images, and the same concepts can be applied to Ophthalmic Plastic Surgery. A number of recent papers have used AI for eyelid and periorbital measurements (91, 92). A similar approach could be used to classify periorbital lesions with 3D stereophotogrammetry and apply AI analysis to aid in diagnosis and triage (93–98).

Lastly, most oculoplastic conditions lend themselves nicely to photographic documentation without specialized camera equipment. This feature can easily be used for electronic medical records and also adopted for transmission via Telemedicine protocols (91). The current pandemic has reinforced the utility of e-consultations, and this trend is bound to continue and expand well beyond this period.

## Author Contributions

The writing of this manuscript was a collective effort among YG, VK, HX, XS, XM, YW, WW, AR, and LH. YG, AR, and LH contributed to the conception and design of the work, the organization of the draft, and the improvement of the manuscript. YG, VK, HX, XS, XM, YW, and WW each drafted initial sections of the paper. All authors contributed to the manuscript revision and approved the submitted version.

## Funding

This work was supported by National Natural Science Foundation of China (Grant No. 82102346).

## Conflict of Interest

The authors declare that the research was conducted in the absence of any commercial or financial relationships that could be construed as a potential conflict of interest.

## Publisher’s Note

All claims expressed in this article are solely those of the authors and do not necessarily represent those of their affiliated organizations, or those of the publisher, the editors and the reviewers. Any product that may be evaluated in this article, or claim that may be made by its manufacturer, is not guaranteed or endorsed by the publisher.
